# Beyond Imaging: Integrating Radiomics, Genomics, and Multi-Omics for Precision Breast Cancer Management

**DOI:** 10.3390/cancers17213408

**Published:** 2025-10-23

**Authors:** Xiaorong Wu, Wei Dai

**Affiliations:** 1 Breast Cancer Unit, Mount Vernon Cancer Centre, White Hill, Northwood, London HA6 2RN, UK; 2 Electrical and Electronic Engineering, Imperial College London, South Kensington Campus, London SW7 2AZ, UK; wei.dai1@imperial.ac.uk

**Keywords:** radiomics, genomics, multi-omics, breast cancer, radiogenomics, personalized treatment, machine learning

## Abstract

**Simple Summary:**

This review explores how radiogenomics improve the understanding and management of breast cancer. Traditional radiomics uses imaging features to describe tumours but often lacks biological context. By integrating imaging with genomic and transcriptomic information, radiogenomics provides deeper insights into tumour biology and supports more precise diagnosis and treatment decisions. The review summarises current applications of radiogenomics in breast cancer to predict breast cancer subtypes, genetic mutations, treatment response, tumour immune environment, and lymph node involvement. It also discusses the methodologies of machine learning in analysing complex data and outlines key challenges such as imaging modalities harmonization, improving reproducibility, and ensuring model interpretability. Overall, this review highlights radiogenomics as a promising step toward more personalised and biologically informed breast cancer care.

**Abstract:**

Radiomics has emerged as a promising tool for non-invasive tumour phenotyping in breast cancer, providing valuable insights into tumour heterogeneity, response prediction, and risk stratification. However, traditional radiomic approaches often rely on correlative patterns of image analysis to clinical data and lack direct biological interpretability. Combining information provided by radiomics with genomics or other multi-omics data can be important to personalise diagnostic and therapeutic work up in breast cancer management. This review aims to explore the current progress in integrating radiomics with multi-omics data—genomics and transcriptomics—to establish biologically grounded, multidimensional models for precision management of breast cancer. We will review recent advances in integrative radiomics and radiogenomics, highlight the synergy between imaging and molecular profiling, and discuss emerging machine learning methodologies that facilitate the integration of high-dimensional data. Applications of radiogenomics, including breast cancer subtype and molecular mutation prediction, radiogenomic mapping of the tumour immune microenvironment, and response forecasting to immunotherapy and targeted therapies, as well as lymph nodes involvement, will be evaluated. Challenges in technical limitations including imaging modalities harmonization, interpretability, and advancing machine learning methodologies will be addressed. This review positions integrative radiogenomics as a driving force for next-generation breast cancer care.

## 1. Introduction

Breast cancer remains the most commonly diagnosed cancer and the leading cause of cancer-related death in women worldwide [1]. In recent years, the advent of novel therapies, particularly targeted treatments and immunotherapy, has significantly transformed the treatment landscape and improved clinical outcomes for patients with breast cancer [2,3,4,5,6]. Despite these advances, treatment response remains highly variable and often unpredictable. A portion of patients do not derive benefit from these therapies, with limited ability to explain or anticipate outcomes. This variability is largely attributed to underlying differences in tumour biology, molecular subtype, and intratumoural heterogeneity [7,8]. Consequently, developing reliable predictive biomarkers is essential to optimize treatment selection and to identify the patients most likely to respond to specific therapies, based on distinct biological characteristics such as tumour subtype and gene mutation. It would not only reduce unnecessary exposure to potentially toxic or ineffective interventions but also help optimise resource allocation and minimise avoidable healthcare costs.

Biopsy with molecular marker is an early biomarker to predict the response of breast cancer to immunotherapy and targeted therapy if the cancer harbouring a particular mutation, such as PIK3CA, ESR, PTEN, AKT, BRCA1/2, or PD-L1 expression [9,10]. However, there are several disadvantages and challenges associated with using biopsy-based molecular profiling as a predictive tool. First, biopsies are typically taken from one accessible lesion for mutation status, which can only represent a small static snapshot of the tumour rather than the full genetic landscape, including other metastatic lesions. It also overlooks the extensive intratumour heterogeneity and rapid molecular evolution of primary or metastatic breast cancer over time. Evidences have shown that the molecular profile of a breast cancer can change over time, especially in response to therapy [11,12]. A mutation present at diagnosis may no longer be relevant at recurrence or progression, making static biopsy data potentially outdated. In addition, even when a mutation is identified, their predictive value is not always clear-cut. Some mutations may have uncertain clinical significance or may not translate to actual treatment response due to downstream pathway alterations or compensatory mechanisms [8,12,13]. Furthermore, studies show that efficacy of immunotherapy is strongly influenced impacted by histopathology, tumour microenvironment (TME), immune contexture, and tumour molecular profiles [14]. Finally, obtaining tissue biopsies is invasive, which may not be feasible in certain cases due to the high risk of procedure complications relating to tumour location or patients’ co-mobilities. Liquid biopsy through circulating tumour cells (CTC) and circulating tumour DNA (ctDNA) analyses is becoming a promising approach for identifying mutation status, such as PIK3CA and ESR, which have sped up treatment decisions [15,16,17]. However, there is a sensitivity issue with low ctDNA level can yield false negative result and clonal haematopoiesis may generate false positives [18,19]. Consequently, a more reliable and non-invasive tool that can predict treatment efficacy in breast cancer alone or in combination with ctDNA or other clinical information is needed to assist clinicians to personalize treatment decisions.

In current clinical practice, the evaluation of breast tumours on mammography, ultrasound, or MRI is predominantly qualitative, relying on quantitative visual assessment and dimensional measurements (e.g., spiculated, rounded, necrotic with microcalcification), density, enhancement pattern, and anatomic relationship to the surrounding tissues to guide treatment decisions. These modalities offer the advantages of accuracy, reproducibility and minimal invasiveness. However, they are limited in their ability to capture the underling biological behaviour of tumours. Imaging alone may not reflect early molecular changes or treatment-induced adaptations, and size-based criteria often fail to distinguish between viable tumour tissue and treatment-related changes such as necrosis, fibrosis, or immune infiltration. As a result, radiological assessment may lag behind actual therapeutic response or resistance at the molecular level. Modern oncology is shifting beyond early diagnosis toward the concept of personalised medicine—delivering the right treatment to the right patient at the right time. Within this paradigm, quantitative analysis of medical images emerges as a natural progression, enabling objective and reproducible biomarkers to reflect changes in tumour metabolism, vascularity, cellularity, or immune activities to support tailored treatment strategies [20]. Recent advances in medical image analysis have been driven largely by artificial intelligence (AI), which allows the processing of vast datasets across imaging modalities. Within this framework, broad methodological approaches have emerged, including radiomics.

Radiomics is an innovative tool with the capability to extract large amounts of measurable information from standard medical images. This information is then analysed using artificial intelligence to find correlations with clinical outcomes, genomic alterations or treatment response. Consequently, treatment strategies can be personalized by taking into the account the specific characteristics of each tumour. AI is a computational system capable of making accurate inferences from a large amount of complex data, primarily through advanced algorithm, namely, machine learning (ML) and, more recently, deep learning (DL) algorithms. Through AI or machine learning, radiomics features can be integrated with clinicopathological data and other multi-omics layers, such as genomics, transcriptomics, or proteomics, to enhance predictive accuracy and support more informed clinical decision-making in personalised breast cancer management. Radiomics has become a powerful tool for non-invasive tumour phenotyping in breast cancer, offering insights into heterogeneity, treatment response, and risk stratification. However, conventional approaches remain largely correlative, linking imaging features with clinical data but lacking direct biological interpretability. Molecular subtyping of breast cancer is crucial for prognostic and predictive purposes due to its diverse clinical behaviours. Radiogenomics, which integrates radiomics with genomics and other multi-omics data, provides an opportunity to personalise diagnostic and therapeutic strategies. By eliminating the need for biopsy and sequencing, it streamlines clinical workflows and contributes to advancing individualized patient care.

**Figure 1:**
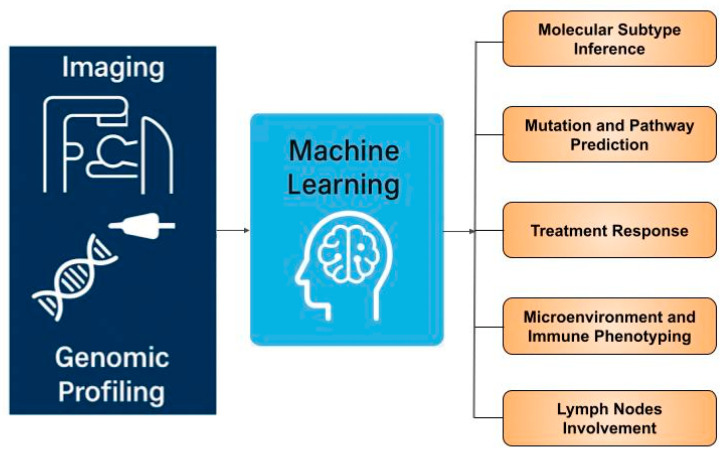
Overview of radiogenomics applications in breast cancer.

While numerous systematic reviews on radiogenomics in breast cancer [21,22,23,24,25,26,27] and other tumour types [28,29] have recently emerged, most primarily focus on methodological aspects—how radiogenomic analyses are performed—rather than their clinical applications or limit to a specific subtopic [24,25,26]. This review emphasizes the translational relevance of radiogenomics in breast cancer, exploring its broad clinical implications, therapeutic potential, and future directions for integration into precision oncology. We highlight advances of radiogenomic applications in breast cancer subtype and molecular mutation prediction, radiogenomic mapping of the tumour immune microenvironment, and forecasting response to targeted and immune therapies, as well as lymph nodes involvement (Figure 1). We also discuss key challenges, including imaging harmonisation, interpretability, and machine learning methodology translation. We aim to provide guidance for clinicians on how to approach and apply this emerging concept.

## 2. Methods

A targeted literature search was performed across PubMed/PMC, ScienceDirect, Google scholar, IEEEXplore, and MDPI using a subset or combination of terms: “radiogenomics”, “radiomics”, “breast cancer”, “breast cancer diagnosis and management”, and “machine learning” specified in the title or abstract. A total of 145 records were initially identified. After removal of duplicates, non-relevant publications, and non-English articles, 22 studies were included in this review.

## 3. Radiomics, Radiogenomics, and Artificial Intelligence

Radiomics offers several distinct advantages in oncology. First, feature extraction from medical images is entirely non-invasive, making it well-suited for routine clinical use with high patient compliance. Second, radiomic analysis captures information from the whole three-dimensional tumour and all associated lesions, providing a more comprehensive assessment of tumour heterogeneity and its microenvironment compared with conventional qualitative imaging evaluation. Third, radiomics allows for longitudinal monitoring, enabling dynamic tracking of tumour changes over the course of treatment and facilitating early detection of treatment resistance [30,31].

Radiogenomics extends the scope of radiomics by linking imaging-derived features with genomic data to uncover the molecular and genetic foundations of breast cancer. While radiomics focuses on quantitative descriptors of imaging phenotypes, radiogenomics integrates these patterns with genomic signatures, thereby offering a biologically informed interpretation of tumour behaviour. The central premise is that imaging phenotypes, derived in vivo and non-invasively, can serve as surrogates for genomic phenotypes captured by transcriptomic profiling. This approach positions radiogenomics at the intersection of systems biology and imaging science, with the potential to refine tumour characterisation, predict therapeutic response, and advance personalised treatment strategies [21].

Artificial intelligence plays a pivotal role in enabling both radiomics and radiogenomics. AI encompasses machine learning and its more advanced subset, deep learning, both of which are designed to emulate human cognitive processes. In radiomic applications, machine learning and deep learning algorithms identify complex, multidimensional patterns within imaging features and generate predictive models that often outperform conventional statistical approaches. A key strength of AI lies in its ability to handle high-dimensional, non-linear data, uncovering subtle associations that may elude human interpretation. Importantly, deep learning methods can learn directly from raw medical images, automatically extracting radiomic features without manual intervention. This capability reduces observer bias, improves reproducibility, and facilitates scalable, standardised image analysis—critical requirements for integrating radiomics into precision oncology [32].

Machine learning in radiomics can be divided into supervised, unsupervised, and reinforcement learning. Supervised learning (e.g., support vector machine, random forest, Cox regression) is most common, linking input features to target outcomes, while unsupervised methods (e.g., K-means, hierarchical clustering) uncover hidden patterns in poorly labelled data. Reinforcement learning uses interactions with the environment to optimize sequential decisions. Deep learning, particularly CNNs (Convolutional Neural Networks) for structured data and standard models for vectorized data, is increasingly applied in medical imaging.

The radiogenomic workflow typically comprises four major steps: image acquisition, segmentation of the Volume of Interest (VOI), feature extraction and selection, and finally model development and validation [31,33]. Model performance is often evaluated using ROC (Receiver Operating Characteristic) curves and AUC (Area Under the Curve), but overfitting from biased or mismanaged training data can inflate results. To ensure generalizability, independent and multi-institution validation datasets are essential [34,35]. Each stage introduces potential sources of variability that can affect the robustness and generalisability of the final predictive model. These technical and methodological challenges partly explain why radiomics has yet to achieve widespread adoption in clinical practice. To illustrate the current state of research, we summarise selected full-text English-language studies on the application of AI-driven radiogenomics in breast cancer in (Table 1 and Table 2).

## 4. Key Applications of Radiogenomics

### 4.1. Imaging and Molecular Subtype Inference

In breast cancer, molecular classification stratifies tumours into five subtypes: luminal A, luminal B, HER2-enriched, basal-like, and normal-like. Each subtype is defined by distinct marker expression patterns and carries a different prognosis [58]. Traditionally, molecular profiling requires biopsy samples from the primary tumour. However, with the increasing use of quantitative imaging techniques such as radiomics, imaging-derived biomarkers in combination of genomics have emerged as non-invasive tools capable of characterising breast cancer molecular subtypes.

Cui et al. [36] analysed 489 breast cancers to determine whether ultrasound radiomic features could predict HER2 status and link to tumour biology. Eight ultrasound radiomic features, primarily GLSZM and GLRLM texture measures, were significantly associated with HER2-positive breast cancer. Zone entropy (ZE) emerged as the key marker, reflecting greater heterogeneity, while the others indicated lower gray-level uniformity, collectively correlating with hypoechoic regions and calcifications seen in HER2-positive tumours. Functional enrichment linked ZE to immune activity and BMP-2 induction, suggesting immune-driven mechanisms of tumour calcification, while associations with oxidative stress point to additional pathways analogous to vascular calcification. These findings provide a biological rationale for ultrasound radiogenomics as a non-invasive predictor of HER2 status. A logistic regression model built from these features achieved an AUC of ~0.80, suggesting moderate predictive accuracy. While the study supports the concept that ultrasound radiomics can serve as a non-invasive surrogate for HER2 status and provide biological insight, it is limited by retrospective design, modest discrimination, and lack of external validation. Larger prospective studies are needed before clinical application.

Instead of USS, Shiri, I., et al. [37] introduced a deep radiogenomics sequencing (DRS) framework using DCE (dynamic contrast-enhanced)-MRI to decode breast tumour gene–phenotype associations. By integrating breast MRI data from 922 biopsy-confirmed invasive breast cancer patients to predict ER, PR, and HER2 status using deep learning networks. Tumour regions were standardised into 3D multi-channel inputs, and models were trained with an 80/20 train–test split. Performance was modest but promising: SEResNet50 predicted ER with AUC 0.695, ResNet34 predicted PR with AUC 0.658, and SEResNext101 predicted HER2 with AUC 0.698. Sensitivity and specificity varied across models. DRS outperformed conventional radiomics in capturing tumour heterogeneity, enabling non-invasive prediction of tumour biology and offering potential to guide precision therapy in breast cancer.

Yoo, J. et al. [38] evaluated whether tumour stiffness, quantified by shear wave elastography (SWE), reflects underlying tumour biology in breast cancer. Results showed that higher SWE-measured stiffness was significantly associated with tumour hypoxia, as well as ER negative/PR negative status, high Ki-67, HER2 positivity, high grade, lymph node metastasis, and more aggressive subtypes (HER2-overexpressing, triple-negative). Shear wave elastography parameters (E_average_ and E_ratio_) significantly correlated with GLUT1 expression in breast cancer. E_average_ was independently associated with GLUT1, suggesting SWE-derived stiffness reflects tumour hypoxia and aggressive biology. These findings suggest that SWE-derived stiffness captures clinically relevant tumour microenvironment features, particularly hypoxia, which may explain its correlation with aggressive tumour phenotypes. SWE thus has potential as a non-invasive imaging biomarker for breast cancer characterization and risk stratification.

There is another pilot study [39] investigated radiomic signatures from hybrid contrast-enhanced ultrasound (CEUS) images to assess the histological characteristics of breast cancer. By combining conventional B-mode ultrasound with CEUS, the researchers extracted multiparametric radiomic features and built predictive models. Key CEUS-derived radiomic signatures showed significant correlations with tumour grade, hormone receptor status (ER, PR), HER2 status, and Ki-67 proliferation index. Radiomic analysis identified wavelet-based features as predictors of receptor status in breast cancer. *WavEnHH_s_4* was independently associated with HER2 positive, while *RWavEnLH_s_4* predicted ER status and a combined model predicted PR status with good specificity. No significant associations were found for Nottingham grade or Ki-67, highlighting the selective value of radiomics for receptor characterization. The results indicate that CEUS-based radiomic profiling can provide non-invasive imaging biomarkers for tumour biology, supporting its potential role in personalized breast cancer diagnosis and treatment planning.

In another study [40], the authors developed a multi-parametric MRI-based radiomics models to predict breast cancer molecular subtypes and androgen receptor (AR) expression. A total of 162 patients with clinical T2–4 stage breast cancer were included. From each pre-biopsy MRI (DCE-T1WI, FS-T2WI, and ADC maps), 4198 radiomic features were extracted, followed by multiple feature selection strategies (LASSO, RFE, mRMR, Boruta, Pearson). Using combinations of feature sets and machine learning classifiers, 120 diagnostic models were developed and tested with LOOCV (leave-one-out cross-validation). The models achieved good performance in distinguishing molecular subtypes and AR status. The findings suggest that MRI radiomics can serve as a non-invasive tool for molecular subtype classification and AR expression assessment, supporting precision treatment planning in breast cancer.

Finally, a large-scale study [41] of 922 breast cancer patients evaluated radiogenomics using 529 quantitative features from preoperative DCE-MRI combined with gene expression profiling. Machine learning models linked radiomic features to molecular subtypes (Luminal A, Luminal B, HER2-enriched, Triple-negative) and key biological pathways. For example, multivariate models showed predictive value for breast cancer subtypes and receptor status, achieving an AUC of 0.697 for Luminal A, 0.654 for triple-negative breast cancer (TNBC), 0.649 for ER status, and 0.622 for PR status. Radiomic signatures showed strong predictive performance, particularly for distinguishing Luminal A vs. non-Luminal A and TNBC vs. non-TNBC. Imaging features related to tumour size, shape, and enhancement heterogeneity correlated with pathways in proliferation, immune regulation, and angiogenesis. In addition, several individual radiomic features were significantly associated with specific subtypes. The robustness of these associations across a large heterogeneous cohort supports the scalability and generalisability of radiogenomics as a non-invasive tool for subtype prediction and biologically informed, personalised therapy planning.

### 4.2. Mutation and Pathway Prediction

As personalised medicine advances, integrating genomic data with imaging features into a unified strategy offers clinicians a more comprehensive understanding of tumour dynamics and enables better-targeted therapies. Alterations in key signalling pathways—particularly the PI3K/AKT/mTOR and MAPK cascades—drive tumour cell survival and proliferation. Radiogenomics provides an emerging approach to bridge these domains, using imaging-derived features to non-invasively predict underlying genetic mutations and pathway activation. By linking radiomic signatures with molecular alterations, radiogenomics holds the potential to identify patients with high-risk tumour biology, stratify them for targeted therapies, and monitor treatment response in real time.

One single-centre prospective study [42] (*n* = 95) correlated pre-treatment MRI features with RNA sequencing profiles across breast cancer subtypes. Specific MRI characteristics, such as mass type and irregular shape, were linked to distinct gene expression patterns: in ER-positive tumours, mass lesions were associated with upregulations of genes related to proliferation and metastasis (CCL3L1, SNHG12, MIR206), and downregulation of tumour suppressors (MIR597, MIR126, SOX17), reflecting poor prognosis, anti-oestrogen resistance, and activation of pathways such as STAT3, while in triple-negative cancers, texture heterogeneity correlated with immune- and extracellular matrix–related gene expression. Increased precontract T1 signal heterogeneity correlated with upregulation of chemoresistance and metastasis genes (CLEC3A, SRGN, HSPG2, KMT2D, VMP1) and downregulation of favourable survival genes (IGLC2, PRDX4). In HER2-positive tumours, early postcontrast T1 enhancement and low T2 signal were linked to proliferation and immune-related gene expression (MLKL, CXCL10), suggesting high angiogenesis, cellularity, and immune infiltration. These results indicate that MRI features can serve as non-invasive surrogates for subtype-specific genetic alterations relevant to prognosis, metastasis, and therapy response. The findings suggest that MRI features can non-invasively capture subtype-specific genetic alterations relevant to prognosis, metastasis, and therapy response, highlighting the potential of radiogenomics for personalized breast cancer assessment.

Ming et al. [43] conducted a multicohort radiogenomic analysis to link DCE-MRI features with gene expression, PAM50 molecular subtypes, and prognosis in breast cancer. Across discovery (*n* = 174) and validation (*n* = 72) cohorts—plus six external prognostic datasets (*n* = 1443)—they identified significant correlations between imaging features and gene expression (e.g., RBP4, MYBL2, LINC00993), notably within the cell cycle pathway. An eight-gene imaging-associated prognostic signature (including CHEK1, TTK, CDC45, BUB1B, PLK1, E2F1, CDC20, CDC25A) robustly stratified outcomes across datasets, with high expression indicative of poor prognosis. Concurrently, machine learning classifiers based on DCE-MRI features accurately predicted receptor status and PAM50 subtypes—ER, HER2-enriched, basal-like, and the prognostic signature—with AUCs ranging from ~0.73 to ~0.84. Additionally, an imaging-only prognostic model directly demonstrated strong survival predictive capacity.

Another multicohort study (*n* ≈ 2500 across four datasets) [44] investigated whether radiogenomic signatures from DCE-MRI could predict Oncotype DX recurrence scores and survival in ER-positive breast cancer. The results demonstrated that radiomic features of tumour heterogeneity and parenchymal enhancement were significantly associated with high versus low recurrence score categories, with AUCs up to 0.89 in validation. Through a radiogenomic mode, patients were successfully stratified into low- and high-risk groups with significantly different outcomes, independent of standard clinicopathological factors. Using an elastic net approach, 11 optimal imaging features (morphologic, first-order, and texture) were identified, achieving a moderate correlation with Oncotype DX recurrence score (RS). Tumours with low sphericity (irregular shape) were linked to higher RS, poorer treatment response, and worse survival, while higher zone entropy—reflecting intratumoural heterogeneity—also correlated positively with RS. Notably, the model also predicted survival in external cohorts without genomic data, underscoring its potential as a surrogate for multigene assays. This imaging-based RS framework therefore offers a scalable, non-invasive alternative to genomic testing, particularly valuable for guiding prognosis and treatment in ER-positive breast cancer patients where tissue assays are unavailable.

In a study performed by Yeh et al. [45], radiomic analysis of preoperative 3T DCE-MRI from 47 invasive breast cancers was integrated with RNA sequencing data. Gene set enrichment analysis of 186 pathways demonstrated that radiomic size features correlated positively with proliferation and negatively with apoptosis. Immune regulation and extracellular signalling pathways showed the strongest associations: tumours with upregulated T-cell receptor, chemokine, cell adhesion, and cytokine–cytokine signalling appeared smaller, more spherical, and texturally heterogeneous. Upregulation of JAK/STAT and VEGF pathways was linked to greater intratumoural heterogeneity, while additional associations were observed with metabolic and catabolic pathways. These findings indicate that DCE-MRI radiomics can non-invasively capture key biological processes in breast cancer, including proliferation, immune signalling, and angiogenesis.

### 4.3. Prediction of Treatment Response

The prediction of response to neoadjuvant chemotherapy (NAC) in breast cancer has emerged as a major focus in radiogenomics research. Integrating baseline images-derived radiomic features with established molecular markers has shown promise in enhancing predictive accuracy. Radiomic analysis captures quantitative information on tumour heterogeneity, shape, texture, and vascularity, providing complementary insights to molecular profiling. Several studies have demonstrated that combining imaging biomarkers with genomic and transcriptomic data improves the identification of patients likely to achieve pathological complete response, enabling more precise treatment stratification and potentially guiding adaptive therapy decisions.

Zhang et al. [46] developed a radiogenomic model to predict pathological complete response (pCR) in TNBC using baseline and early-treatment DCE-MRI features combined with genomic mutation data. In 112 patients receiving neoadjuvant chemotherapy, radiomics-only models (based on light gradient boosting machines) yielded AUCs of ~ 0.71–0.73. Incorporating variant allele frequencies from core biopsies significantly improved performance—the radiogenomic model reproduced an AUC of 0.87 in the validation cohort. They identified a series of wavelet-related radiomics features that contributed to pCR prediction. Additionally, the MED23 p.P394H mutation was identified as a potential therapeutic resistance marker: it conferred Epirubicin resistance in vitro, likely via altered homologous recombination repair (through the *p*-ATM–*γ*-H2A.X–*p*-CHK2 axis). These results support the use of integrated radiogenomic signatures to enhance early prediction of treatment response and to uncover actionable resistance mechanisms in TNBC.

A similar study [47] was carried out by Zhou group, who developed a preoperative radiogenomic multiscale model integrating MRI-based tumour heterogeneity features with genomic data to predict both pathological complete response and prognosis in triple-negative breast cancer patients receiving neoadjuvant chemotherapy. The integrated radiogenomic–pathology model (genomics–pathology–radiomics model, GPRM), captured spatial and hemodynamic heterogeneity, achieved the highest accuracy, and effectively stratified survival outcomes, supporting its role as a non-invasive predictive biomarker for personalized treatment planning. The models identified genomic alterations (e.g., MED23, REL) linked to poor response and demonstrated prognostic value in stratifying disease-free survival. These findings highlight the utility of multiscale, multi-omics integration for precision treatment planning in TNBC.

Lai, J., et al. [48] conducted a multi-cohort study stratifying breast cancer patients into low- and high-risk groups using rad-score, gene-score, and nomogram predictions. The analysis indicated that immunotherapy may be more effective in the high-risk group. Drug sensitivity assessment revealed differing IC50 values between risk groups. Notably, the high-risk group exhibited elevated expression of PD-L1, CD8A, CTLA4, CXCL10, GZMB, and HAVCR2, suggesting a potentially stronger response to targeted immunotherapies. Risk stratification based on the nomogram also demonstrated significant differences in PD-1 expression. These findings highlight the clinical relevance of distinguishing high- and low-risk patients, as such stratification can guide personalized drug regimens to optimize treatment outcomes in breast cancer.

In a retrospective study [49], Oda, S. and team integrated MRI radiomics with microarray gene expression data to predict pathological complete response in breast cancer patients receiving neoadjuvant chemotherapy. The combined radiogenomic model outperformed radiomics or genomics alone, achieving higher predictive accuracy for pCR and demonstrating the potential of integrative multi-omics approaches for individualized treatment planning. This study demonstrated that radiomics features (e.g., wavelet-based root mean squared and cluster shade, reflecting intratumoural heterogeneity and textural asymmetry) were predictive of poor treatment response, consistent with prior evidence linking heterogeneity to resistance. In the genomic model, transcriptional regulation, protein synthesis, and chromosome maintenance genes (e.g., GTF2I, SMC3, EIF3A) were top contributors, reflecting their roles in proliferation and genome stability. In the radiogenomic model, gene expression features dominated, while MRI-derived features became less influential, though some remained consistently predictive. The integration of radiomic and genomic data identified novel predictors and improved pathological complete response prediction, highlighting the complementary value of a radiogenomic approach. Larger cohorts and external validation are needed to confirm these findings.

### 4.4. Tumour Microenvironment and Immune Phenotyping

The tumour microenvironment—comprising stromal cells, extracellular matrix components, hypoxia, and angiogenesis—plays a central role in resistance by activating growth factor signalling pathways and diminishing drug efficacy. Adaptive cellular processes such as autophagy and epithelial–mesenchymal transition (EMT) further enhance resistance, allowing cancer cells to survive therapeutic stress [59]. A comprehensive understanding of these mechanisms is critical for improving therapeutic strategies and patient outcomes. In this context, radiogenomics offers a powerful, non-invasive approach to characterising the tumour microenvironment and immune phenotypes by linking imaging features with molecular pathways. Such integrative methods can provide insights into immune infiltration, stromal interactions, and signalling activity, thereby advancing precision approaches to treatment response prediction and resistance management.

Fan et al. [50] analysed tumour–stroma heterogeneity (TSH) in breast cancer by integrating gene-expression and DCE-MRI radiomic features. A cellular TSH biomarker stratified patients into favourable and poor survival groups, with good-survival patients showing stronger tumour–stroma correlations in cytotoxic lymphocyte abundance, reflecting enhanced immune surveillance and antitumour activity. Radiomic features linked to TSH—including IDMN, flatness, and sphericity—were used to construct a radiogenomic signature, which was independently validated in two cohorts (*n* = 180 and *n* = 61). Higher IDMN values and stromal uniformity predicted favourable outcomes, while higher tumour flatness and sphericity correlated with poor survival. A composite TSH-derived radiogenomic score correlated significantly with overall survival and recurrence-free survival retaining independent prognostic value after adjustment for clinicopathological factors. Importantly, this imaging-based approach enables longitudinal, non-invasive assessment of tumour–stroma and immune dynamics, extending prior radiomic–immune studies and offering robust prognostic markers for risk stratification in breast cancer.

Instead of MRI, Dou and colleagues [51] conducted a radiogenomic analysis linking ultrasound-derived tumour characteristics and clinical features with immune-related gene expression in breast cancer. Their findings demonstrated that certain sonographic phenotypes, such as tumour stiffness and vascularity, are significantly associated with immune gene signatures, suggesting that imaging features may serve as non-invasive biomarkers of the tumour immune microenvironment. For example, CXCL2, MIA, NR3C2, PTX3, S100B, SAA1, SAA2, and CXCL9 genes were correlated with each other and with clinical and ultrasonic characteristics. They also mentioned that high MIA expression was associated with PR positivity, while low NR3C2 expression correlated with larger tumour size (≥20 mm), later stage, HER2 positivity, and high Ki-67 (≥20%). NR3C2 also showed negative correlations with PKI and AUC on contrast-enhanced ultrasound and positive correlations with AT and TTP. PTX3 expression was negatively correlated with PKI and Emax from shear wave elastography. SAA2 expression was linked to the presence of burrs on tumour edges, and CXCL9 expression was associated with age of onset and tumour stage. This work highlights the potential of ultrasound-based radiogenomics to provide insights into tumour–immune interactions and to support the development of predictive tools for immunotherapy responsiveness.

### 4.5. Axillary Lymph Node Involvement

Axillary lymph node metastasis (ALNM) is a key prognostic factor in breast cancer and plays a critical role in treatment planning. Accurate preoperative prediction of ALNM can guide surgical decision-making and help avoid unnecessary axillary dissections. Recent advances in radiogenomics, combining imaging features with genomic data, offer a non-invasive approach to stratify patients by metastatic risk. Integrating multi-modal data has the potential to improve predictive accuracy and support personalized management strategies in breast cancer care.

As previously mentioned in the multicohort study conducted by Lai, J., et al. [48], they also integrated MRI-derived radiomics with whole-transcriptome sequencing to develop a radiogenomic machine learning model for predicting axillary lymph node metastasis in breast cancer. This retrospective study analysed 1,078 breast cancer patients from TCGA, TCIA, Duke, and Foshan cohorts. A support vector machine was used to screen radiological and genetic features, and a multimodal model integrating radiogenomic and clinicopathological data was developed to predict ALNM. The integrated radiogenomic nomogram achieved strong predictive performance in the TCIA cohort distinguished ALN negative from ALN positive patients. Risk subgroup analysis revealed significant differences in pathway enrichment and immune checkpoint expression between high- and low-risk groups, suggesting distinct therapeutic sensitivities. The radiogenomic multimodal nomogram, which incorporates imaging, genomic, and clinicopathological data, can accurately predict ALNM and potential treatment responses in breast cancer patients, supporting its value for precision clinical decision-making.

Another multicohort study [52] integrated MRI features with transcriptome data to develop and validate a radiogenomics model for predicting axillary lymph node metastasis in breast cancer. This retrospective study combined transcriptomic data with matched MRI data for 111 breast cancer patients, serving as the training and testing cohorts, while an additional 15 patients from a separate hospital formed the external validation cohort. Feature selection and dimensionality reduction were performed using Boruta, and logistic regression was employed to develop genomics, radiomics, and integrated radiogenomics models to predict axillary lymph node metastasis. The radiogenomics model, incorporating five genomics and three radiomics features demonstrated good predictive performance. The model, using a stepwise logistic regression model with both direction, five genomics features and three radiomics features, was validated across cohorts, and key genes linked to metastasis (e.g., PTPN21, ST6GALNAC3, FAM13A, CHRNA7) enriched in PI3K/Akt and Notch/mTOR pathways contributed to prediction. Findings highlight the potential of radiogenomics to noninvasively predict nodal status and guide treatment planning.

## 5. Limitation, Current Challenges, and Future Directions

### 5.1. Limitations

Despite substantial progress, most these radiogenomic studies in breast cancer remain retrospective and single-centre in nature. These studies are typically constrained by heterogeneity in imaging acquisition, segmentation, and feature extraction, which introduces methodological bias and limits reproducibility. Especially, inconsistent imaging parameters, lack of voxel intensity normalisation, and diverse dimensionality reduction strategies contribute to instability in radiomic features and hinder the development of generalisable models. To address these issues, greater standardisation and harmonisation are essential. Establishing consensus on acquisition parameters and preprocessing protocols—including voxel intensity normalisation and segmentation criteria—would substantially reduce technical variability across studies. Transparent documentation of feature selection processes and open sharing of annotated datasets are equally important, given the absence of a universally optimal feature selection algorithm. These steps would facilitate reproducibility, enable independent validation, and be key to achieving clinically robust and generalisable radiogenomic models for future application in breast cancer.

Furthermore, adequate sample size is essential for developing robust and generalisable radiogenomic models. Most radiogenomic studies in breast cancer are limited by small cohort sizes and frequently rely on public datasets, primarily due to the restricted availability of matched genomic data. Small, underpowered sample sizes are prone to overfitting and false-positive associations, thereby limiting model reproducibility and clinical translation. This limitation can be alleviated through improved access to shared imaging and genomic database, as well as data augmentation strategies that expand training datasets via rotating or inverting the images, or more advanced techniques such as generative adversarial networks (GANs), which can synthetically generate realistic cross-modality images. In parallel, multicentre collaborations are crucial for increasing data heterogeneity, enhancing statistical power, and enabling rigorous external validation across diverse populations and imaging platforms.

Besides these methodological considerations, strengths and limitations of different imaging modalities, as well as technical approaches and model development strategies also represent key technical and translational challenges and suggest strategic directions to advance radiogenomics toward clinical implementation.

### 5.2. Modality-Specific Challenges

Various imaging modalities are routinely incorporated into the diagnostic workup of breast cancer. In recent years, there has been growing scientific interest in radiomic biomarkers derived from these modalities to improve diagnosis and treatment planning. Mammography remains the most widely used screening tool and is effective for early detection; however, its sensitivity decreases in women with dense breasts or prior surgical interventions. Advanced techniques—such as contrast-enhanced mammography (CEM), contrast-enhanced spectral mammography (CESM), and digital breast tomosynthesis—have expanded the radiomics applications in this domain. To improve screening performance, ultrasound is often combined with mammography, though it carries a high false-positive rate.

Ultrasound has shown additional potential, particularly with the development of advanced acquisition methods such as elastography, Doppler imaging, and contrast-enhanced ultrasound (CEUS), which provide functional information on tissue properties. Breast MRI is now an established diagnostic and preoperative staging tool, offering the advantage of avoiding ionizing radiation and enabling multiparametric acquisitions. These include morphological data from T1- and T2-weighted sequences, along with functional insights from dynamic contrast-enhanced MRI (DCE-MRI) and diffusion-weighted imaging (DWI-MRI).

Beyond breast-focused imaging, whole-body techniques such as PET-CT and whole-body MRI are increasingly applied to provide comprehensive staging, including axillary involvement and distant metastases, thereby supporting more personalized therapeutic decisions. More recently, hybrid imaging approaches have gained attention for their potential to integrate multiparametric information, although most studies to date have focused on correlating radiomic features with immunohistochemical breast cancer data.

Integrating these diverse imaging datasets into radiogenomic frameworks presents significant opportunities to capture tumour heterogeneity, link phenotypic features to underlying molecular profiles, and develop predictive models for prognosis, treatment response, and therapy stratification. This multimodal imaging foundation sets the stage for constructing robust radiogenomic models, highlighting the need for sophisticated feature extraction, selection, and machine learning approaches to translate imaging-derived data into clinically actionable insights.

### 5.3. Technical and Modelling Challenges

The final stage of radiogenomic involves developing mathematical models that generate predictive information and address specific research objectives through machine learning algorithms to uncover complex patterns within the data. Machine learning shares considerable overlap with classical statistics, as many of its foundational principles originated in statistical theory. However, the two fields diverge in their goals and evaluation strategies. In ML, model performance is typically assessed by predictive accuracy or related metrics, whereas statistical models are traditionally evaluated by goodness-of-fit to a specified model. A notable distinction is the rarity of formal hypothesis testing in ML research. Clinical studies usually rely on frequentist hypothesis testing, where a significance level (α, often 0.05) is chosen, and a p-value is calculated to determine whether observed results would occur by chance under the null hypothesis. By contrast, ML prioritises building models that maximise predictive performance, often using prior information or exploring model spaces, as in Bayesian approaches, rather than inferring belief between competing hypotheses. This difference reflects the greater complexity of ML applications. In breast cancer radiogenomics, a wide range of machine learning approaches have been explored, each offering distinct advantages and limitations. Several studies [40,42,43] have compared common machine learning models such as support vector machine (SVM), logistic regression (LR), random forest (RF), multilayer perceptron (MLP) models, etc. to assess their predictive performance in radiogenomics. However, no single model consistently outperforms others across different datasets, highlighting that performance depends on study design and feature selection. Combining multiple models or using ensemble approaches may improve robustness. More recently, researchers have explored novel modelling strategies to overcome these limitations and achieve better generalization.

Liu and Hu [53] explored the radiogenomic associations between deep learning-derived MRI features, genomic profiles, and clinical characteristics in breast cancer. They use a stacked convolutional denoising autoencoder (DA), which is an unsupervised deep learning model to extract intrinsic features from data. The unsupervised approaches to extract feature in a data-driven manner have a higher flexibility of representing data intrinsic patterns than supervised hypothesis-driven methods as a supervised model will force the features to just represent the label information used to train the model, instead of representing data intrinsic patterns. Their findings suggest that deep radiomics features (DRFs) extracted from MRI can reflect molecular alterations and clinically relevant subtypes, offering a potential avenue for non-invasive tumour characterisation and personalised treatment. For example, the DRFs “fea_4043” is significantly associated with 55 breast cancer risk genes, two gene signatures, and 89 KEGG pathways.

Furthermore, Chen, L., et al. [54] proposed a Conditional Probabilistic Diffusion Model (CPDM) to generate synthetic radiogenomic data in breast cancer. Conventional radiogenomic studies require imaging, genomic, and clinical data from the same cohort, which is often unfeasible. CPDM addressed this limitation by generating synthetic MRIs from multi-omics inputs (gene expression, copy number variation, DNA methylation) for 726 TCGA-BRCA patients lacking imaging data. The model produced high-quality images with strong predictive performance, accurately classifying ER+/HER2+ subtypes and patient survival outcomes. Notably, CPDM performed robustly despite being trained on relatively small datasets, a strength attributed to its probabilistic framework that models full distributions rather than single best-fit solutions. This approach assigns low probability to outliers and high probability to samples fitting the training distribution, enhancing prediction reliability. Feature simplification (e.g., grayscale imaging, focusing on task-relevant regions of sparse MRI data) further improved efficiency. By integrating MRI features with transcriptomic profiles, the model captured cross-modal relationships and produced biologically plausible synthetic data. Overall, CPDM demonstrates the potential of diffusion-based generative models to overcome data scarcity, expand radiogenomic applications, and accelerate precision oncology research in breast cancer.

Another group [55] introduced a novel integrative computational framework for radiogenomic biomarker discovery in breast cancer by combining MRI-derived radiomic features with transcriptomic data. The approach uncovered imaging–genomic associations capable of stratifying patients by prognosis and therapeutic response, demonstrating that multimodal integration provides greater predictive power than either modality alone. Methodologically, the framework employed Bayesian Tensor Factorization (BTF) to identify biologically meaningful multi-genomic features associated with cancer hallmarks, while deep learning was applied to automatically extract radiomic features, enhanced with explainability tools and domain knowledge. Importantly, a leveraging strategy was proposed to address the challenge of unpaired imaging–genomic data in DL-based radiogenomics. DL-derived radiomic features outperformed traditional approaches, and several prognostic biomarkers were identified, including one potentially linking natural killer cell function to survival. These biomarkers, being non-invasive and clinically feasible, illustrate how integrative radiogenomics can capture both morphological and molecular heterogeneity, reduce reliance on biopsies, and advance precision oncology in breast cancer.

The IMAGGS (image–gene–gene set) study [56] presents a radiogenomic framework designed to uncover multi-way associations between imaging and genomic data in breast cancer subtypes. Using a dataset of 721 patients, each with 12 multi-angle ultrasound images and gene mutation profiles, IMAGGS integrates radiomic and genomic features to identify complex many-to-many relationships, moving beyond traditional one-to-one correlations. The framework screens key radiomic and genomic features and applies gene set enrichment analysis to explore the joint biological effects of gene sets in breast cancer subtypes. IMAGGS was further validated on a glioblastoma dataset, demonstrating its scalability across diseases and modalities. Overall, IMAGGS enables comprehensive characterization of tumour phenotypes and genotypes, supporting biological interpretation and potential targeted therapy development.

Another radiogenomics study [57] developed an omics-to-omics joint knowledge association subtensor (JoKAS) model to integrate genomic data with ultrasound imaging in breast cancer radiogenomics. Unlike traditional approaches that examine one-to-one associations between specific ultrasound features and individual cancer genes, the JoKAS framework applies subtensor factorization to uncover many-to-many cross-modal modules, capturing complex relationships across omics and imaging. To address tumour heterogeneity, the model incorporates a sparse weighting strategy that identifies subgroup-specific associations, thereby distinguishing global from context-dependent patterns. When applied to breast cancer cohorts, JoKAS revealed clinically meaningful imaging–genomic signatures linked to tumour heterogeneity and subtype differentiation. Compared with conventional single-modality or pairwise methods, the model achieved greater accuracy in predicting clinical outcomes and biological features. These findings underscore the promise of subtensor-based integration for discovering robust radiogenomic biomarkers and advancing precision oncology.

For clinical translation of radiomics, model reproducibility and generalizability are crucial. Validation can be internal (e.g., K-fold cross-validation, bootstrapping) for model tuning, or external (e.g., temporal or geographic validation using independent datasets) to ensure unbiased performance and real-world applicability. External validation is especially important to avoid feature selection bias and to account for variations in imaging protocols and patient demographics. Tools such as the Radiomic Quality Score (RQS) and reporting standards, alongside a phased framework for study design, aim to enhance study quality, transparency, and the eventual adoption of radiomics in healthcare [60].

Future directions in breast cancer radiogenomics are expected to align closely with advances in precision therapeutics and AI-driven clinical decision-making. As radiogenomic models mature, their ability to non-invasively infer molecular alterations such as PIK3CA, ESR1, and HER2 mutations could inform therapeutic decision-making and support the use of next-generation inhibitors and combination treatment strategies. Moreover, integrating radiogenomic signatures with genomic, pathological, and clinical data through advanced computational and AI-driven frameworks may enable dynamic patient stratification, identifying subgroups likely to benefit from specific targeted or immune-based therapies. Incorporating radiogenomic endpoints into prospective clinical trials will be critical to validate their predictive and prognostic value, facilitating the translation of these models into routine clinical workflows and personalised treatment planning.

Emerging molecular technologies such as liquid biopsy, proteomics, and spatial transcriptomics are increasingly viewed as complementary to radiogenomics, offering orthogonal insights into tumour biology. Liquid biopsy enables longitudinal monitoring of tumour evolution and therapeutic resistance through circulating tumour DNA and cells, providing a dynamic molecular perspective that can be correlated with imaging-based phenotypes. Proteomics extends radiogenomic interpretation by capturing post-translational modifications and protein-level dynamics that underpin treatment response. Spatial transcriptomics directly links gene expression to tissue microarchitecture, creating an opportunity to biologically validate imaging-derived features and enhance mechanistic interpretability. Integrating these modalities with radiogenomic and AI-driven frameworks represents a promising direction for developing holistic, multi-scale models of tumour behaviour, facilitating more precise patient stratification and guiding rational therapeutic combinations in breast cancer management.

## 6. Conclusions

Radiogenomics in breast cancer has achieved key advances, including associations with molecular subtypes and mutational pathways, predictive models for treatment response, and integration with genomic profiles that link imaging features to tissue architecture. However, clinical translation remains constrained by the need for methodological standardization, larger and more representative cohorts, mechanistic validation through spatial multi-omics, and prospective trials demonstrating utility. Moving toward routine adoption will require coordinated multicentre studies, transparent and reproducible pipelines, and pragmatic trial designs to establish radiogenomics as a reliable tool in breast cancer management.

## Figures and Tables

**Table 1:** Summary of selected literature on radiogenomics in breast cancer.

Reference	Applications	Size	Images	Results	Data Sources
[36]	HER2	489	USS	GLSZM and GLRLM predicted HER2 positive	In-house
[37]	ER PR HER2	922	DCE-MRI	SEResNet50 predicted ER, ResNet34 predicted PR, and SEResNext101 predicted HER2	Public data
[38]	Subtype Ki-67 Grade Lymph node	82	USS/SWE	SWE stiffness predicted tumour aggressiveness (hypoxia, ER-/PR- status, high Ki-67, HER2 positivity, high grade, lymph node metastasis)	In-house
[39]	ER PR HER2 Ki-67	72	CEUS	WavEnHH_s_4 predicted HER2+, RWavEnLH_s_4 predicted ER	In-house
[40]	Subtype AR	162	DCE-MRI	predict molecular subtypes and AR status	In-house
[41]	Subtype	922	DCE-MRI	Imaging heterogeneity correlated with proliferative, immune, and angiogenic activity	In-house
[42]	Gene pathway	95	MRI	MRI features predict subtype-specific genetic alterations relevant to prognosis, metastasis, and therapy response	In-house
[43]	PAM50 Subtype	174	DCE-MRI	Eight-gene prognostic signature	Mixed
[44]	Oncotype DX RS response	130	DCE-MRI	11 predicted Oncotype Dx score	In-house
[45]	Gene pathway	47	DCE-MRI	Radiomics reflects proliferative, immune, and angiogenic processes	In-house
[46]	pCR in TNBC	112	DCE-MRI	wavelet-related radiomics features predicted pCR	In-house
[47]	NACT response in TNBC	98	DCE-MRI	Intratumoural and peritumoural feature with genomic predict response and prognosis in TNBC	Mixed
[48]	ALNM treatment response	1078	MRI	radiogenomic multimodal nomogram predict ALNM and treatment responses	Public data
[49]	NACT response in TNBC	112	DCE-MRI	wavelet-based root mean squared and cluster shade, reflecting intratumoural heterogeneity and textural asymmetry predicted poor treatment response	In-house
[50]	TSH	332	DCE-MRI	Radiomic features linked to TSH—including IDMN, flatness, and sphericity	Public data
[51]	Immune-related gene	38	USS	CXCL2, MIA, NR3C2, PTX3, S100B, SAA1, SAA2, and CXCL9 genes were correlated	In-house
[52]	ALNM	111	DCE-MRI	radiogenomics model, incorporating five genomics and three radiomics features demonstrated good prediction of ALNM	Mixed
[53]	Staging and risk genes	110	MRI	DRF kernels are related to grow factors and risk genes	Public
[54]	Subtype and survival	58	MRI	The model can accurately classify ER+/HER2+ subtypes and patient survival outcomes.	Public date
[55]	Gene pathway		DCE-MRI	Prognostic biomarkers predicted natural killer cell function to survival	Public data
[56]	Subtype	721	USS	IMAGGS can identify subtypes	In-house
[57]	Gene profile	777	USS	tumour heterogeneity and subtype differentiation	In-house

Abbreviations: GLSZM (Gray Level Size Zone Matrix); GLRLM (Gray Level Run Length Matrix); URFs (Ultrasound Radiomics Feature); DCE (dynamic contrast-enhanced); SWE (shear wave elastography); AR (androgen receptor); pCR (pathological complete response); TNBC (Triple Negative Breast Cancer); NACT (neoadjuvant chemotherapy); ALNM (Axillary Lymph Nodes Metastases); TSH (tumour stroma heterogeneity); IDMN (Inverse Difference Moment Normalized); DRF (Deep Radiomic Feature); IMAGGS (image–gene–gene set).

**Table 2:** Summary of selected radiogenomics in breast cancer.

Reference	Segmentation	Non-Radiomics Features	Validation	Model
[36]	CNN-based	RNA expression	Internal validation	Logistic regression
[37]	three-dimensional bounding boxes	Transcriptomic data	Internal validation	Deep radiogenomics sequencing (DRS) model
[38]	manually	GLUT1 expression	unknown	Spearman correlation and logistic regression
[39]	manually	pathology	unknown	Fisher coefficients and univariate analysis for feature selection. Multivariate analysis for prediction
[40]	manually	Gene expression profile	leave-one-out cross-validation	multiple feature selection strategies (LASSO, RFE, mRMR, Boruta, Pearson), Multiple diagnostic models
[41]	Auto	Pathology	Internal validation	Multivariate models
[42]	manually	RNA sequencing	unknown	Unknown
[43]	Auto	RNA sequencing PAM50	Internal and external validation	LASSO embedded logistic regression for feature selection ENR, SVM, RF and naïve bayes NB for model.
[44]	manually	Oncotype RS score	Internal validation	LASSO and ridged model for feature selection. Multivariable Cox proportional hazards model for prediction
[45]	Auto	RNA sequencing data	unknown	
[46]	Semiauto	Genomic mutation data	Internal validation	LASSO with XGBoost for features selection (Light-GBM for prediction)
[47]	manually	Whole genetic data	Internal validation	Univariate analysis and correlation analysis for feature selection plus LASSO and Logistic regression to finalize the feature selection logistic regression for model
[48]	manually	whole-transcriptome sequencing	External validation	Univariate analysis and correlation analysis for feature selection and SVM for feature selection Multimodal model nomogram from rad-score and gene-score
[49]	manually	Gene microarray analysis	Cross-validation	three models (radiomics, genomics and radiogenomics) based random forest
[50]	manually	Gene expression profile	External validation	Cross-validation-based feature selection Standard statistics for prediction
[51]	manually	Immune related gene expression	Internal validation	Spearman correlation
[52]	manually	transcriptomic data	External validation	Boruta method including Univariate analysis and correlation analysis for feature selection Logistic regression for model
[53]	unknown	Genomic profiles	Internal validation	LASSO Denoising autoencoder (DA)-unsupervised DL model
[54]	Auto	mRNA gene expression, DNA methylation, and copy number variation	Internal validation	largest CV for feature selection CPDM for prediction
[55]	Unknown	Transcriptomic data	Internal validation	BTF and deep learning model
[56]	Unknown	gene mutation profiles	Internal and external	machine learning algorithm rAdaSMCCA that implemented feature selection
[57]	manually	Genomic data	internal	omics-to-omics joint knowledge association subtensor model

Abbreviations: CNN (Convolutional Neural Networks); LASSO (Least Absolute Shrinkage and Selection Operator); RFE (Recursive Feature Elimination); mRMR (maximum relevance–minimum redundancy); ENR (Elastic Net Regression); SVM (support vector machine); RF (random forest); Light-GBM (light gradient boosting machine); CV (Coefficient of Variation); CPDM (Conditional Probabilistic Diffusion Model); BTF (Bayesian Tensor Factorization).

## Data Availability

No new data were generated or analysed in this study.
